# Cardiopulmonary exercise testing – A new addition to pre-anaesthetic armamentarium

**DOI:** 10.4103/0019-5049.68367

**Published:** 2010

**Authors:** SS Harsoor, Zulfiquar Ali

**Affiliations:** Editor, Indian Journal of Anaesthesia, #21, 2^nd^ Cross, Kirloskar Colony Basaveshwar Nagar, 2^nd^ Stage, Bangalore, India. E-mail: harsoorss@hotmail.com; 1 Department of Anaesthesia and Intensive Care, Government Medical College, Srinagar - 190 011, India

Cardiovascular complications are important causes of morbidity following major non-cardiac procedures.[1] Risk stratification of these patients, needed during preoperative assessment, often relies on non-invasive tests for myocardial ischaemia. It is essential to understand that pre-operative risk assessment and pre-operative optimization of cardiac disease are performed jointly. The effectiveness of preoperative interventions in high-risk patients undergoing major surgery is undeniable; the patients’ co-morbid conditions can be improved prior to surgery and even the decision for surgery can be modified as a result of cardiopulmonary exercise testing (CPX test).[2]

Many strategies have been devised for pre-operative risk assessment, the earliest being the ASA grading devised by the American Society of Anaesthesiologists (ASA) in 1963. Goldman *et al*.[3] published an article, ‘Multifactorial Index of Cardiac Risk in Non-cardiac Surgical Procedures’. It added some objectivity to the assessment of the patient, by taking into account few of the surgery-specific risks.

Cardiopulmonary exercise testing (CPET or CPX), a non-invasive technique, provides an integrated assessment of cardiovascular and pulmonary function, both at rest and under stress. It can be used to test the ability of the subject’s physiological response to cope with the metabolic demands created by the trauma of major surgery.

A subject is exposed to incremental physical exercises up to a maximally tolerated level. Physiological variables, which include, ventilator parameters, inspiratory and expiratory gases, blood pressure (BP) and electrocardiogram (ECG) are recorded. From these are derived two key indicators: the body’s maximum oxygen uptake (VO_2_max) and the point at which anaerobic metabolism exceeds aerobic metabolism, known as Ventilatory Anaerobic Threshold (AT or VAT); these are noted. They indicate the ability of the cardiovascular system to deliver oxygen to the peripheral tissues and the ability of the tissues to utilise that oxygen.

## BASIC PHYSIOLOGICAL PRINCIPLES

Lactate accumulation occurs in the exercising muscle, when the oxygen demand of the muscle exceeds the supply. At this point the venous lactate concentration starts rising and this point is called the Lactate Anaerobic Threshold (LAT).

The muscle lactate / pyruvate ratio increases at the LAT, which supports the concept that lactic acidosis results from relative muscle hypoxia.[4–6]

**Figure 1:**
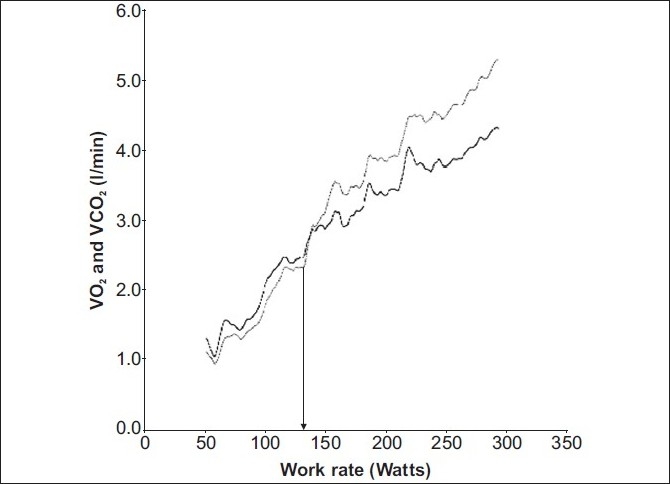
VO_2_ and VCO_2_ vs work rate: VO_2_ and VCO_2_ initially increase linearly and proportionately to the work rate in early exercise. A point is reached where VCO_2_ increases and exceeds VO_2_ (arrow). Towards maximum exercise VO_2_ reaches a plateau while VCO_2_ continues to rise

**Figure 2:**
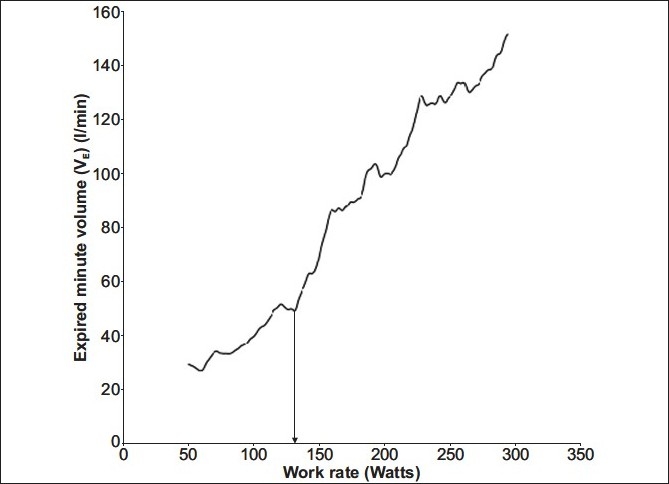
Expired minute ventilation vs work rate. The VAT occurs when the VE slope begins to steepen (arrow) during an incremental exercise test, indicating an increase in VE due to carotid body stimulation, as a result of lactate accumulation

**Figure 3:**
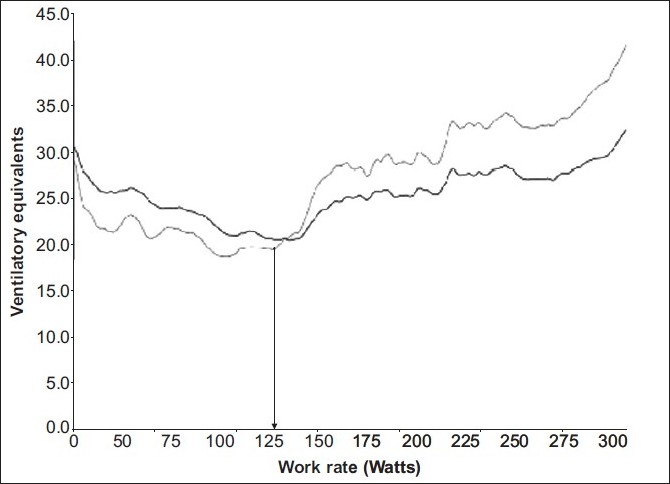
Determining AT by ventilatory equivalents of O_2_ (VE / VO_2_) and CO_2_ (VE / VCO_2_): with incremental work the VE / VO_2_ (light black curve) and VE / VCO_2_ (dark black curve) initially decrease to reach a plateau. The AT is determined by marking the point (arrow) at which VE / VO_2_ starts to increase, whereas, VE / VCO_2_ remains constant or falls slightly (due to VE increasing disproportionately to VO_2_ but proportionately to VCO_2_). The work rate at which AT occurs can then be derived (arrow)

As an individual commences an incremental exercise test, the expired minute volume (VE), oxygen consumption per minute (VO_2_) and CO_2_ production per minute (VCO_2_) increase linearly with respect to variables such as work rate or time. However, a point is reached when VCO_2_ increases out of proportion to VO_2_ [Figure 1]. This change is attributed to HCO_3_ buffering the lactate produced and consequently generating a relative excess of CO_2_. This transition is termed as the Lactate Anaerobic Threshold (LAT). This phenomenon allows the determination of the anaerobic threshold (AT) by respiratory measurements. Generally this is seen at 47 – 64% of VO_2_max in healthy untrained individuals,[7] and is a marker of the maximum work rate that can be sustained for a prolonged period.[8,9] On reaching the AT a fall in the arterial pH, caused by anaerobic respiration, results in the carotid bodies stimulating an increase in minute ventilation.[10] This point is termed as the Ventilatory Anaerobic Threshold (VAT) [Figure 2]. During this period the VE increases in proportion to the VCO_2_ and the VE / VCO_2_ remains constant. The rate of the rise in VE exceeds the rate of the rise in VO_2_, and therefore, VE / VO_2_ starts to increase. This is one method of determining the VAT from gas exchange measurements, as AT is the point where the Ventilatory Equivalent for oxygen (VE / VO_2_) increases relative to a constant Ventilatory Equivalent for carbon dioxide (VE / VCO_2_) [Figure 3].

## CARDIOPULMONARY EXERCISE TESTING IN PRACTICE

There are several different protocols for achieving VO_2_max. Most use either an exercise bicycle (cycle ergometer) or a treadmill. Cycle ergometers used in peri-operative clinical settings provide a degree of safety in maximal testing, offset the work of walking in the obese and are less prone to artefacts in the electrocardiogram (ECG), airflow and pressure measurement.

In both methods, the work rate is gradually and imperceptibly increased in a stepped manner until the subject is unable to continue. On a treadmill this is achieved by increasing both the gradient and speed, whereas, on an exercise bicycle it is achieved by increasing the resistance of the pedals while the subject maintains a constant pedalling rate. The optimal duration of the exercise is approximately 10 minutes.[11] Too short a duration of exercise may produce an underestimation of VO_2_max, while too long a duration may cause premature cessation due to the subject becoming demotivated. VAT seems to be independent of the duration of exercise.[11]

## CURRENT APPLICATIONS OF CPET

The statement of the American Thoracic Society /American College of Chest Physicians on Cardiopulmonary Exercise Testing (2003) lists the specific indications for CPET testing.[12]

1. Evaluation of exercise tolerance, where the diagnosis is known, in order to objectively evaluate functional capacity, disability, or response to treatment.
2. Evaluation of undiagnosed exercise intolerance where cardiac and respiratory aetiologies coexist, the symptoms are disproportionate to the results of resting investigations or the investigations are non-diagnostic.
3. Evaluation of patients with cardiovascular diseases.
4. Evaluation of patients with respiratory diseases/ symptoms.
5. Pre-operative evaluation.
6. Exercise evaluation and prescription for pulmonary rehabilitation.
7. Evaluation of impairment / disability.
8. Evaluation for lung, heart and heart–lung transplantation.

In the current issue of IJA, the authors Milind Bhagwat and Kagerre Paramesh discussed in detail the use of CPET in pre-operative assessment and its use in delineating patients who were to be shifted to intensive care units or wards.

## CARDIOPULMONARY TESTING AS A PRE-OPERATIVE PREDICTOR OF RISK

A review of multiple lung tumour resection studies by Beckles *et al*.[13] provides data to suggest that VO_2_max can be used to predict peri-operative complication rates in tumour resection surgery:

1. If >20 ml/kg/min = no increased risk of complications or death.
2. If <15 ml/kg/min = increased risk of peri-operative complications.
3. If <10 ml/kg/min = a very high risk of post-operative complications. In a clinical study it was observed that the amount of oxygen uptake, exercise ability and cardiac function, during exercise, decrease in patients of lung cancer with tumour vascular invasion depending on the number of invaded vessels.[14]

Cardiopulmonary testing is used to assess the urgency of cardiac transplantation, by comparing the survival of heart failure patients who were not accepted for cardiac transplantation versus those who received a transplant.[15] For those not accepted for transplantation, a VO_2_ peak > 14 ml/kg/min yielded survival comparable to those after transplantation; a VO_2_ peak < 10 ml/kg/min yielded lower survival.

However, few studies have investigated the role of CPET in the pre-operative assessment of patients undergoing non-cardiopulmonary thoracoabdominal surgery. If CPET is able to provide a reliable indication of peri-operative morbidity and mortality it may have a significant impact on the care of surgical patients and on the cost of their healthcare. By identifying high-risk candidates, additional resources can be put in place ahead of time (e.g., an Intensive Care bed) to ensure appropriate post-operative care, and thus potentially reduce morbidity and mortality. Furthermore, if candidates identified as being at risk are placed on an exercise regime before elective surgery this can potentially improve their CPET measurements, and in turn their post-operative risk.

In cardiac surgery, and some forms of pulmonary surgery, it is expected that cardiac and respiratory function will improve post-operatively. In contrast, after non-cardiothoracic surgery, the cardio-respiratory function will remain, at best, unaffected, and may well deteriorate post-operatively, at a time when the demands placed on the cardio-respiratory system are increased. Hence, CPET can play a major role in the pre-operative assessment of these patients. However, with regard to techniques, a large number of randomised studies and a meta-analysis of several randomised clinical trials in non-cardiac surgery patients, comparing the outcome with regional and general anaesthetic techniques, has shown little consistent evidence of improved outcome and reduced post-operative morbidity and mortality.[16]

## FUTURE

To gain further evidence to support the use of CPET as a predictor of post-operative morbidity and mortality, further studies need to be conducted. These studies need to focus on several key areas, particularly: (i) Further investigation of the physiology of AT and the clinical relevance of this phenomenon; (ii) Can other variables independently or combined with VO_2_max and AT provide a more powerful index? (iii) Can the risk threshold of the variables be refined for different types of major surgery? (iv) Does increasing a subject’s VO_2_max or AT pre-operatively significantly improve their post-operative outcome? (v) What, if any, is the significance of the variables recorded during the recovery phase of the test?
